# Emerging Cationic Nanovaccines

**DOI:** 10.3390/pharmaceutics16111362

**Published:** 2024-10-25

**Authors:** Ana Maria Carmona-Ribeiro, Yunys Pérez-Betancourt

**Affiliations:** 1 Biocolloids Laboratory, Departamento de Bioquímica, Instituto de Química, Universidade de São Paulo, São Paulo 05508-000, Brazil; 2 Department of Microbiology, University of Chicago, Cummings Life Science Center 920 E 58th St., Chicago, IL 60637, USA; ybetancourt@bsd.uchicago.edu

**Keywords:** cationic lipids and polymers, nanoadjuvants, nanovaccines against infectious diseases, nanovaccines against cancer, immunotherapies, nanoparticles from self-assembly, immunological cell death

## Abstract

Cationic vaccines of nanometric sizes can directly perform the delivery of antigen(s) and immunomodulator(s) to dendritic cells in the lymph nodes. The positively charged nanovaccines are taken up by antigen-presenting cells (APCs) of the lymphatic system often originating the cellular immunological defense required to fight intracellular microbial infections and the proliferation of cancers. Cationic molecules imparting the positive charges to nanovaccines exhibit a dose-dependent toxicity which needs to be systematically addressed. Against the coronavirus, mRNA cationic nanovaccines evolved rapidly. Nowadays cationic nanovaccines have been formulated against several infections with the advantage of cationic compounds granting protection of nucleic acids in vivo against biodegradation by nucleases. Up to the threshold concentration of cationic molecules for nanovaccine delivery, cationic nanovaccines perform well eliciting the desired Th 1 improved immune response in the absence of cytotoxicity. A second strategy in the literature involves dilution of cationic components in biocompatible polymeric matrixes. Polymeric nanoparticles incorporating cationic molecules at reduced concentrations for the cationic component often result in an absence of toxic effects. The progress in vaccinology against cancer involves in situ designs for cationic nanovaccines. The lysis of transformed cancer cells releases several tumoral antigens, which in the presence of cationic nanoadjuvants can be systemically presented for the prevention of metastatic cancer. In addition, these local cationic nanovaccines allow immunotherapeutic tumor treatment.

## 1. Introduction

Both microparticles (MPs) and nanoparticles (NPs) in efficient vaccines deliver antigens and immunomodulators to cells in the lymphatic system specialized in antigen presentation [1].

Particle traffic to the draining lymph-nodes (LN) depends on particle size; microparticles (500–2000 nm) mostly become associated with dendritic cells (DC) from the injection site whereas NPs (20–200 nm), including virus-like particles VLP (30 nm), are found both in LN-resident DC and macrophages; in vivo imaging studies in mice demonstrate that DC are strictly required for the transport of MPs from the injection site to the LN in contrast to the free drainage of NPs to the LN [2,3,4,5,6].

Biodegradable MPs of poly (lactide-co-glycolide) with a cationic surface adsorb DNA and improve its delivery to DC after intramuscular injection [7]; MPs grant protection for DNA carried over 2 weeks in vitro and increase the DNA translation in vivo. After i.m. immunization, MPs not only significantly enhance serum antibody responses in comparison to unprotected DNA but also cellular immune responses at a low dose of the cationic component [8,9]. The delivery of DNA vaccines using biodegradable polymeric magnetic NPs as opposed to MPs are evidence that DNA vaccines with NPs as carriers are preferentially taken up by dendritic cells inducing maximal levels of combined humoral and cellular immunity [10].

The comparison between MPs and NPs was recently reviewed for the NPs and MPs of aluminium salts [11]. MPs are very polydisperse in water dispersions (sizes varying from 500 up to 10000 nm of mean diameter) inducing improved humoral immune responses; importantly, nanometric sizes are shown to change these adjuvants to yield cellular immune responses [12,13]. For cancer immunotherapy, Al-NPs can elicit a robust and durable balanced (Th1/Th2) immune response and increase the number of cytotoxic T cells compared to Al-MPs, thereby inducing lysis of the cancer cells [11]. Nanosystems are worth studying for one main reason: they are directly driven to the lymph nodes to stimulate the dendritic cells whereas the microparticles remain at the site of injection exerting a depot effect and depending on macrophages to reach the lymphatic system [2].

**Figure 1:**
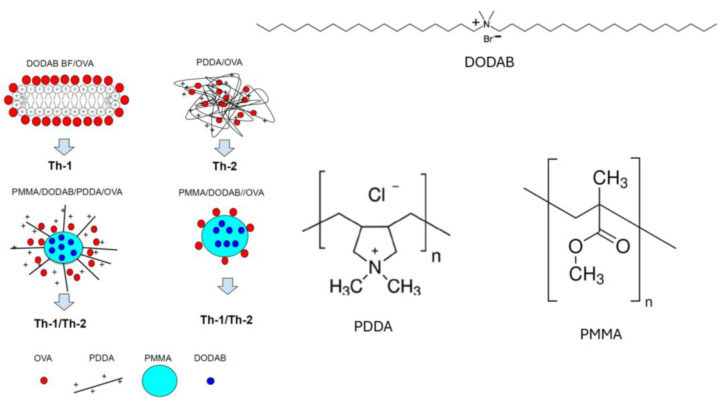
Immune responses Th-1 and/or Th-2 elicited by cationic nanostructures carrying ovalbumin (OVA) depend on affinity between OVA antigen and adjuvant. Molecules used to build the nanostructures were: the synthetic lipid dioctadecyl dimethyl ammonium bromide (DODAB), the polyelectrolyte PDDA and the biocompatible polymer poly (methyl methacrylate) (PMMA). Reproduced from reference [14,16].

Regarding NP charge, the cationic ones electrostatically combine with oppositely charged antigens or immunostimulants of interest such as peptides, proteins, nucleic acids, oligonucleotides, etc. [14,15,16,17,18,19,20,21,22]. At a molecular level, cationic compounds such as lipids, surfactants and polymers [14,23,24,25,26,27] have been used to build cationic nanostructures and can elicit different immune responses depending on the affinity between the antigen and the adjuvant. For cationic NPs adsorbing the model antigen ovalbumin (OVA), four different cationic nanostructures were evaluated regarding the occurrence of humoral and cellular responses as illustrated in Figure 1. This figure was reproduced from reference [14].

The cationic NPs were based on the four different compositions. Combined with OVA, the cationic bilayer fragments and the cationic polymer elicited Th1 and Th2 immune responses, respectively. The other two cationic nanostructures yielded balanced Th1/Th2 responses (Figure 1). The affinity between OVA and the cationic polymer yielded entangled nanostructures from which OVA could not easily be released to the DC cytosol for presentation via major histocompatibility complex one (MHC-1); OVA degradation in the endosome would be required in order to expose OVA derived peptides by MHC-2. On the other hand, OVA loosely attached to cationic bilayer fragments which possibly bypassed the endosome, easily detaching from the adjuvant in the cytosol for presentation by MHC-1. A high affinity between antigen and adjuvant was implied in the Th-2 response whereas a low affinity was implied in the Th-1 response. Intermediate affinities in the other two cationic nanostructures shown in Figure 1 yielded both responses [16]. NP shape has also been shown to be important [18,25,26,28,29,30,31]. For example, whereas micrometric liposomes of the cationic lipid dioctadecyldimethylammonium bromide (DODAB) as adjuvants carrying a recombinant protein of *Mycobacterium leprae* induced a large cellular (Th-1) immune response and poor humoral response [26], the use of nanometric DODAB bilayer fragments (DODAB BF) as adjuvants to carry outer membrane antigens of *Neisseria meningitidis* produced a balanced cellular (Th-1) and humoral response (Th-2) [32].

In this review, the adjuvant properties of cationic nanostructures are overviewed in the context of vaccines to prevent or treat infections and cancer. In the next section, the role of size and positive charge in vaccines design is discussed.

## 2. Effects of Size and Charge of the Adjuvants

NP size significantly influences DC uptake, which is crucial for effective vaccine delivery and the initiation of an immune response. Endocytosis was defined as the main mechanism for cell uptake of NPs smaller than 500 nm in diameter whereas capture by macrophages occurred for particle sizes above 500 nm [31]. In the lungs, alveolar macrophages ingested more efficiently liposomes of 0.65–2 μm than smaller ones [33,34].

DC are able to promote differentiation of CD4+ T cells toward Th1 or Th2 subsets according to the different invaders of the host organism [35]. The pro-inflammatory cytokines, such as IFN-γ and TNF-α, are secreted by Th-1 cells which eliminate invaders by cytolysis. Th-2 cells secrete preferentially anti-inflammatory cytokines, such as interleukins 4, 5, 6 and 13 for activating the humoral immune responses destined to avert the pathogen.

NPs about 2 nm in diameter do not activate DC due to their diffusion into and out of cells and their non-membranous intracellular localization, whereas NPs 12 nm in size enter DCs by receptor mediated-endocytosis and elicit a Th1 response: this was depicted from incubation of DC in culture with the 12 nm gold NPs and the determination of CD80 and CD86 that are markers of DC activation; also IFN-γ, IL-10 and IL-12 secretion profiles confirmed the inflammatory profile for the 12 nm gold NPs [36]. A major review on effects of the role of size, shape and rigidity on immune responses of nanovaccines in general reference [37] is suggested.

OVA presented by 50 nm polymeric NPs contrasted to micrometric MPs for OVA presentation regarding the immune responses elicited in mice shown to be Th1 for the nanometric particles [3,38] and Th2 for the micrometric ones [39,40].

Similar associations between size and type of immune response were also found for fungi [41]. The respiratory tract of humans maintains sterilizing immunity against fungi; the smaller conidia of fungi drive respond toward Th1 in contrast to hyphae that induce Th2 [42]. Curiously, nano-spikes on TiO_2_ MPs yielded Th1 responses in spite of the micrometric size of the particles [43].

NPs around 20–50 nm are particularly effective for receptor-mediated endocytosis, which is a selective and efficient method for internalizing materials at the cellular level. This size range allows NPs to interact optimally with cell surface receptors, which facilitates the encapsulation of the nanoparticles within cellular vesicles and their subsequent direct release into the cytosol for processing and presentation to T cells [44,45].

Particles within the nanoscale are readily internalized by cells via endocytosis, a process that encompasses various mechanisms including phagocytosis, macropinocytosis, and receptor-mediated endocytosis. The efficiency and pathway of uptake are highly dependent on the NPs’ size [31,44,46].

Larger NPs, typically those over 100 nm, tend to be internalized via phagocytosis or macropinocytosis. These pathways are less selective and involve the engulfment of particles along with extracellular fluid, which can lead to less efficient antigen presentation. The larger size of these particles may result in their accumulation within endosomal or lysosomal compartments, potentially leading to the degradation of the encapsulated antigens rather than their processing and presentation on MHC molecules [47,48].

Conversely, smaller nanoparticles, particularly those under 20 nm, can diffuse more freely across cellular membranes but might bypass the endocytic pathway, leading to the suboptimal delivery of antigens to the immune system. This can reduce the immunogenicity of the vaccine, since antigens can fail to reach the cellular compartments where they are needed to elicit a strong immune response. Such small NPs would freely enter or leave the cell, thereby decreasing the total vaccine efficacy, as observed experimentally [48,49]; they also are excreted by the kidneys [49,50].

Optimizing NP size for vaccine delivery ensures antigen internalization into the cytoplasm of target cells, processing and presentation in a manner that maximizes the immune response. This optimization directly influences the efficacy of vaccines, particularly those designed to induce cellular immunity, critical for fighting intracellular pathogens and cancer cells.

Once delivered into the cytoplasm, antigens are processed into peptides through the proteasome degradation pathway, reach the endoplasmic reticulum and bind to MHC class I molecules for exposure at the cell surface and recognition by T-cell receptors on cytotoxic T lymphocytes (CTLs), which, upon activation, proliferate and differentiate into effector cells capable of targeting and destroying cells presenting the same antigenic peptides [1,51,52].

NPs can be engineered to release antigens slowly, promoting sustained immune engagement and the development of long-lasting immune memory. This controlled release, combined with the particles’ ability to mimic the size and shape of pathogens, enhances their uptake by dendritic cells, leading to more effective T-cell activation [53]. This approach not only maximizes the vaccine’s immunological impact but also facilitates dose sparing, important in scenarios where vaccine availability is limited.

The lymphatic vessels, primarily responsible for transporting fluids, cells and other substances, have an endothelial lining that allows particles of certain sizes to pass through more effectively [54]. Particles in the range of 20–100 nm are ideal because they are small enough to enter the lymphatic capillaries, yet large enough to avoid rapid clearance by renal filtration. NPs sized between 20 and 100 nm are optimally designed for lymph node targeting, as their dimensions allow them to enter the lymphatic system through interstitial spaces and travel efficiently to lymph nodes [49,50]. The NPs’ ability to target lymph nodes and facilitate dendritic cell maturation and antigen presentation is illustrated by the development of a manganese-based nanodriver (PLHM). For glioblastoma suppression, the PLHM system efficiently stimulates interferon genes with the production of type I interferon and the enhancement of the immune response against the tumor; the incorporation of doxorubicin in the NPs further amplifies this effect, demonstrating a potent synergistic interaction that enhances tumor suppression [55].

Lipid nanoparticle-mediated lymph node-targeting delivery of mRNA vaccines showcases significant advancements in cancer immunotherapy, particularly through the enhancement of CD8+ T cell responses. Novel lipid NPs, named 113-O12B, were designed to target lymph nodes directly without the need for active targeting ligands. NPs demonstrated a superior ability to deliver mRNA encoding full-length ovalbumin (OVA) and TRP-2 peptide antigens directly to lymph nodes, leading to significantly enhanced CD8+ T-cell activation compared to conventional lipid NPs. NPs’ targeting capability resulted in potent therapeutic and protective effects against melanoma models, particularly the B16F10 melanoma model. Importantly, the study found that 113-O12B NPs, when used in conjunction with anti-PD-1 therapy, showed a complete response in a substantial proportion of cases, underscoring the potential of this approach to substantially improve cancer vaccine efficacy. The effective delivery of mRNA to DC in lymph nodes not only minimized side effects associated with non-targeted delivery systems but also maximized the immunogenic potential of the vaccines by enhancing antigen presentation directly where it is most effective for initiating robust immune responses [56].

The positive charge on cationic NPs is typically conferred by the presence of amine groups [57,58,59] or quaternary ammonium moieties [60,61,62,63]. Particularly important are the cationic lipids or surfactants such as DODAB and cetyltrimethylammonium bromide (CTAB), respectively [64,65,66,67], and some hydrophilic polymers such as PDDA, PEI, PLGA, PMMA and chitosan [68,69,70,71,72,73] given the broad range of sizes available for producing cationic NPs from combinations of DODAB and silica or DODAB and polymers over a range of controllable nanometric sizes [74,75]. The cationic nature of these NPs enhances their interaction with negatively charged biological molecules and cellular membranes, making them particularly effective for delivering genetic material and antigens into cells. Their positive charge facilitates strong interactions with negatively charged cellular membranes, which improves cellular uptake of the vaccine components such as antigens and other immune stimulators [76]. Furthermore, loaded mRNA and protein antigens in cationic NPs become protected from degradation by enzymes in the extracellular environment. Combinations of mRNA and cationic lipid NPs protect mRNA from degradation by RNAase as demonstrated by mRNA analysis using agarose electrophoresis; there was also a superior antigen loading capacity [77,78]. This protective effect increases the likelihood that a higher quantity of intact antigen reaches the target cells, enhancing the potential for a strong immune response [79]. Combinations between cationic lipids and polymers also allow interesting developments such as the cationic supported bilayers on polystyrene sulfate nanoparticles [80] or the hybrid cationic lipid-biocompatible polymer NPs with the polymer selected as PLGA or PMMA [66,81,82].

By facilitating the delivery of antigens directly into the cytoplasm [83,84], cationic NPs enable the antigens to be processed and then exposed by MHC-1 complex [84]. This pathway activates CD8+ cytotoxic T lymphocytes, which are critical for killing infected cells and tumor cells. The ability to enhance this pathway is particularly beneficial in vaccines aimed at eliciting strong cellular immunity, as required against intracellular bacteria and cancer. Additionally, the interaction of cationic NPs with immune cells can lead to the activation of various toll-like receptors (TLRs), which play a critical role in the innate immune system [59,85]. Cationic NPs act similarly to danger signals coming from infective agents such as agonists of molecular patterns recognition receptors like lipopolysaccharides and lipoteichoic acids from bacteria.

NPs’ positive charge can induce the activation of NALP3 inflammasome; this occurs via the NPs’ interaction with cellular components that trigger the inflammasome pathway [57,59,86,87]. Working with macrophage cells in culture, cationic lipid elicited NF-κB, TNF-α, IL-1β, IL-6 and IFN-B by human or mouse macrophages. Further, there was the activation of inflammatory cascades by the cationic lipid RPR206252. Cationic lipid also activated the Toll-like receptor 2 (TLR2), and NOD-like receptor pyrin 3 (NLRP3), the main component of the inflammasome [57,59]. Moreover, inherent properties of cationic NPs can lead to the rupture of cell membranes [20,84,88,89]. This disruption releases intracellular contents that are recognized as damage-associated molecular patterns (DAMPs). These DAMPs then stimulate further immune responses, attracting more immune cells to the site of vaccine administration [84].

Similarly to cationic NPs, assemblies of cationic lipids in vivo are potent inducers of pro-inflammatory cytokines such as those required for the cellular immune response and combat of intracellular pathogens [18,21,26,90]. The use of cationic lipids in vivo is limited not only by their pro-inflammatory properties but also by their poor stability [91,92]. In vitro, there is a strong affinity between serum proteins and cationic lipid carriers driven by electrostatic attraction and the hydrophobic effect [20,93,94]. In vivo, the interaction of the cationic carrier with albumin and immune globulins leads to opsonization and rapid removal of the protein-surrounded cationic carriers from the circulation by the macrophages of the reticule endothelial system (RES) [95]. Thus, most formulations based on cationic nano-carriers are delivered subcutaneously, intradermally, intratumoral or via intraperitoneal routes in order to avoid the inflammatory toxicity and the elimination by RES associated to the cationic carriers administered by the intravenous route [96]. In order to disguise the inflammatory properties of cationic lipid assemblies and their cargo we employed a dual strategy to obtain controllable inflammation: the anti-inflammatory drug indomethacin complexed with the cationic lipid carrier was encapsulated by carboxymethylcellulose (CMC) [97]; this approach still requires further evaluation in vivo for antigens delivery by the intravenous route. Similarly, the anti-tubercular drug rifampicin was complexed with cationic lipid nano-assemblies and the complexes surrounded by CMC and delivered in vitro to micobacteria achieving 100% reduction in micobacteria cell viability [98]; these assemblies should be further tested in vivo aiming at simultaneous drug and vaccine delivery against micobacteria.

Cationic NPs can also facilitate the escape of encapsulated antigens from endosomes and lysosomes to the cytosol. By destabilizing the membranous barriers of endosomes, the NPs prevent the degradation of antigens, ensuring their proper processing and presentation on MHC class I molecules, which is essential for the activation of CD8+ T-cells [99,100,101]. Lysosomal escape enhances the presentation of antigens to the immune system, leading to a more robust activation of T-cells [102]. The ability of cationic NPs to bypass the degradation pathways and present antigens directly to the immune system is a pivotal factor in their effectiveness as vaccine adjuvants [103]. The activation of the immune system by cationic NPs through these mechanisms—NALP3 inflammasome activation, cell membrane rupture and lysosomal escape—highlights their potential as powerful tools in vaccine development. These properties not only enhance the immediate immune response but also contribute to the long-term efficacy of vaccines by ensuring the generation of memory cells [104,105,106].

The physicochemical properties of cationic NPs—such as colloidal stability, spontaneous interaction with negatively charged antigens, enhanced cellular uptake and adjuvant effects—make them exceptionally useful in the development of efficient and effective vaccine formulations. Stability is relevant for effective immunization strategies. Firstly, the colloidal stability of these nanoparticles ensures that they remain dispersed in solution, preventing aggregation that could diminish their efficacy and safety. This stability is particularly important for maintaining the integrity and uniformity of vaccine formulations during storage and transport [15,20,21]. Furthermore, the versatility of cationic nanoparticles allows for the co-delivery of antigens and adjuvants within the same particle. This co-delivery can synchronize antigen and adjuvant presentation to the immune system, thus maximizing the immune response [21,107].

**Figure 2:**
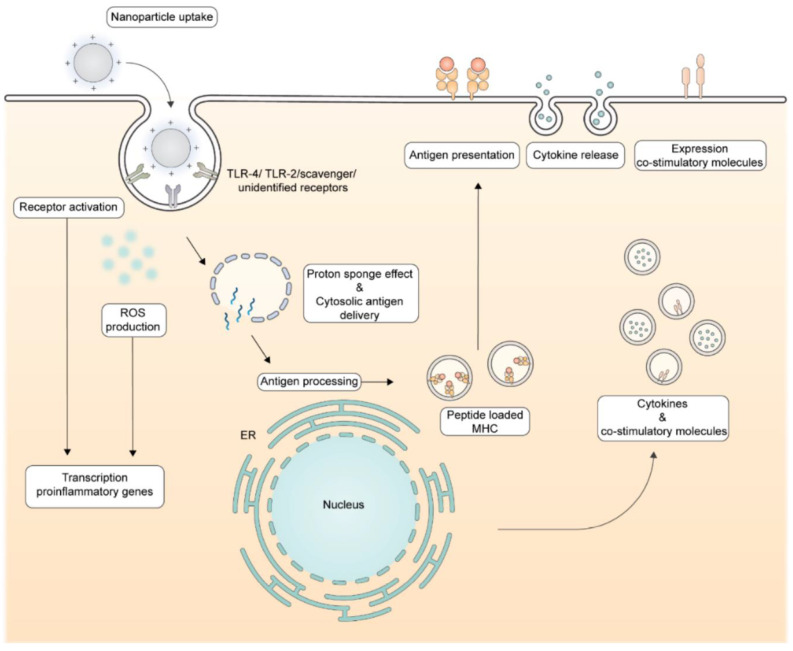
Intracellular routes followed by cationic nanoparticles (NPs) acting as immunoadjuvants. NPs induce production of free radicals (ROS), activate receptors (e.g., TLR-4) and improve transcription of pro-inflammatory genes with production of specific cytokines determining inflammation. Endosomal rupture releases antigen to the cytosol where the antigen starts to be processed to yield smaller peptides to be presented as such by the major histocompatibility complex (MHC) after transportation to the surface of the cell. Improving antigen release in the cytosol induces proliferation of antigen-specific T-cells sub-sets. Reproduced from [79].

Figure 2 illustrates the routes followed by cationic NPs upon their interaction with dendritic cells (DC) that culminate in antigen presentation to the immune system.

In our laboratory, cationic NPs and assemblies still need further testing as adjuvants for vaccines. Among them some examples are the supported cationic bilayers on silica [22,108,109,110] or polystyrene sulfate [19,65,74,75,80], the hybrid lipid-polymer NPs based on the layer-by-layer approach [111,112] or on the interaction forces driving formation of the cationic lipid-biocompatible polymer nanostructure [15,82,113] or cationic polymer-biocompatible polymer NPs [16,82,114] or on the electrostatic attraction between cationic polymer and antigen [20]. The bilayer fragments (BF) made of synthetic lipids such as the anionic sodium dihexadecyl phosphate (DHP) [115] or the cationic DODAB [116] were first described in the eighties and nineties and have been used in drug and vaccine delivery since then [18,97,117,118]. DODAB BF are one of the most explored nanostructures (60–80 nm) for carrying a variety of antigens [21,24,28,119]. In this review, we discuss seminal and recent advances in applications of cationic nanostructures such as immunoadjuvants for vaccine design against infectious diseases and cancer.

## 3. Cationic Nanostructures in Vaccine Design Against Infections

Cationic nanosystems are worth studying for three main reasons: (1) they efficiently combine with oppositely charged antigens such as nucleic acids, negatively charged peptides and proteins and others; (2) they activate the inflammasome pathway and increase antigen uptake by APCs; (3) they protect biodegradable antigens in vivo [76].

**Figure 3:**
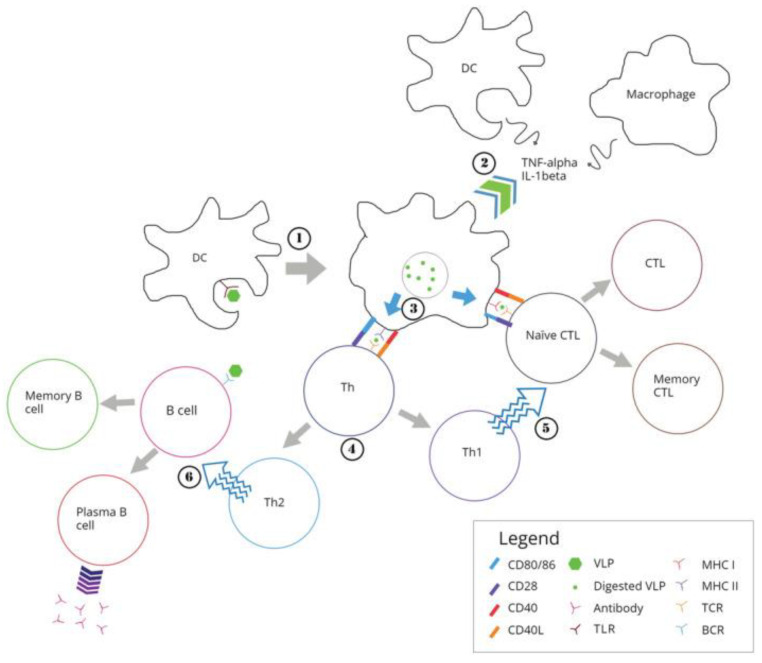
Virus-like particles (VLPs) and the immune system. In **step 1**, interaction with receptors on the dendritic cell (DC) and VLP uptake. In **step 2**, maturation of DC and release of cytokines such as TNF-α and IL-1β that are pro-inflammatory with recruitment of additional antigen presenting cells (APCs), including DC and macrophages. In **step 3**, enzymatic hydrolysis of VLP antigens into peptides with their binding to MHC I and MHC II for presentation at the DC surface. In **step 4**, peptide on MHC II, CD40 (in red) and CD80/86 (in blue) interact with T-cell receptor TCR, CD40L (in orange) and CD28 (in magenta) present on naïve helper T-cell (Th), respectively, followed by proliferation and differentiation of Th cells into type 1 (Th1) and type 2 (Th2) cells; in addition, peptides on MHC I of the DC, CD40 and CD80/86 also interact with TCR, CD40L, and CD28 present on the naïve cytotoxic T lymphocyte (CTL). On **step 5**, aided by Th1, naïve CTL proliferates and differentiates into effector and memory CTLs for cellular immunity. On **step 6**, naïve B cell interacts with VLP (from the blood stream) or DC via B cell receptor (BCR). With help of Th2, the B cell differentiation into plasma B cells occurs; the plasma B cells account for active release of antibodies in humoral immune response; in addition, differentiation into memory B cells also takes place, being responsible for the long-lasting antibody production. Reproduced from reference [121]. Copyright (2022), with permission from John Wiley and Sons.

Against viral infections, biomimetic NPs [110,119,120] and virus-like NPs (VLP) used in the design of vaccines mimic native viruses but lack viral genomes being similarly taken up by the immune system [121]. Figure 3 illustrates the processing of VLPs by the immune system [121].

DNA manipulation using recombinant DNA technology has been intimately associated with our understanding of microorganisms including the pathogens that cause many infectious diseases in humans [122]. The scaling-up of antigen production for subunit vaccines design can readily be achieved from techniques that enable us to read, copy and manipulate DNA sequences.

**Figure 4:**
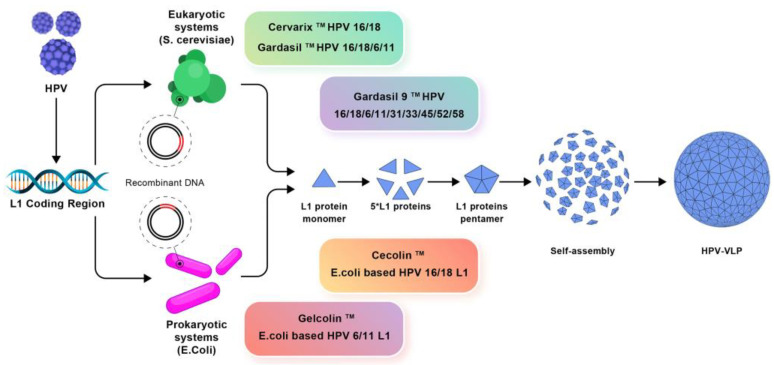
The amplification of L1 coding region of human papilloma virus (HPV) using recombinant DNA technology for production of prophylactic vaccines. One should notice that the self-assembly of the L1 recombinant protein generated the virus-like nanoparticle (HPV-VLP). Reprinted from reference [123].

A good example is the development of vaccines against the human papilloma virus (HPV) as illustrated on Figure 4: three prophylactic HPV vaccines based on the recombinant DNA technology were approved and purified L1 protein self-assembled as HPV empty shells and induced production of specific neutralizing antibodies [123]. One should notice that not all antigens self-assemble to yield NPs in dispersion. In many cases the vaccine subunit design requires also adjuvant(s) and/or immunostimulator(s).

The technique of DNA cloning signaled the birth of genetic engineering, which allowed the facile transfer of genes among different biological species [124]. Thereby, genes encoding important recombinant proteins such as insulin and growth hormone were cloned and expressed in *Escherichia coli* or *Saccharomyces cerevisae*; hundreds of pharmaceuticals such as therapeutic proteins and antibodies, including the monoclonal ones, became available in the market produced by the biotechnology industry [125]. Recombinant DNA technology indeed represents a valuable tool also in vaccines development which contributed to the fast design of COVID-19 vaccines applied during the recent pandemics as discussed next.

**Figure 5:**
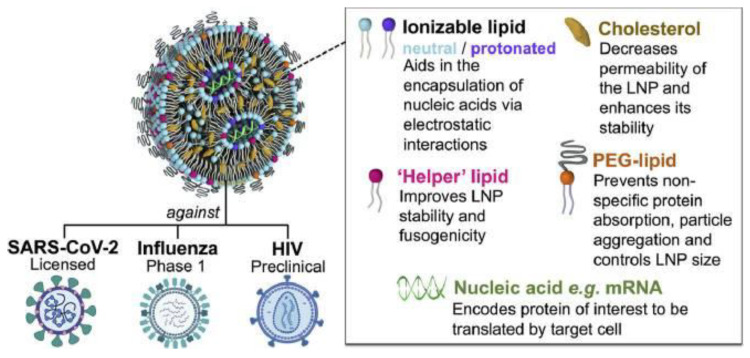
Cationic lipid nanoparticle mRNA vaccines applied to several infectious diseases. Reproduced from reference [128]. Copyright (2021), with permission from Elsevier.

Cationic NPs also played a fundamental role in the delivery of mRNA vaccines, as highlighted by their successful use in the COVID-19 vaccination [126,127]. Encapsulation of mRNA into cationic lipid nanoparticles not only hampers premature degradation but also enhances its uptake into cells, primarily through endocytosis facilitated by the positive charge of the cationic NPs [128,129]. Figure 5 illustrates the design of the cationic lipid nanoparticles (LNP) used to encapsulate the mRNA molecules and protect them from degradation by nucleases before reaching their target cells [128].

The coronavirus spike protein (S) has been widely used for vaccine development; prospective vaccines used strategies such as virus inactivation, live-vectoring, nucleic acid and recombinant antigen vaccines [130]. For recombinant vaccine development some approaches have been producing DNA-based vaccines by cloning the SARS-CoV-2 S-protein; using the DNA plasmid-carrying SARS-CoV-2 S gene; using the S protein mRNA; and by employing virus-like particles mimicking SARS-CoV-2 lacking the virus genetic material. Vaccines targeting the S protein sensitize the host inducing cellular and humoral immune responses accompanied by the desired immunization [130].

Tuberculosis (TB) has surpassed AIDS globally [131]. In 2022, TB was responsible for 1.3 million deaths, including 167,000 people with HIV [131,132]. Caused by the bacterium *Mycobacterium tuberculosis*, TB in the lungs can affect other organs in the body [132]. The disease is particularly prevalent in low-income countries, exacerbating public health and economic disparities [131]. Currently, the only widely used vaccine, Bacillus Calmette-Guérin (BCG), has been in use since 1921; although BCG is effective in preventing severe forms of TB in children, its efficacy in adults, particularly against pulmonary TB, which is the most common form and the main source of transmission, is variable and generally poor [133,134]. In this context, cationic adjuvants present a promising avenue for enhancing TB vaccine efficacy.

The efficacy of cationic liposomes, with or without cholesterol, was tested in various compositions as delivery systems for TB antigens; the goal was to identify cationic lipids with strong immunogenic properties and understand how cholesterol modifies the formulation’s efficacy; cationic liposomes were prepared using the thin-film hydration method followed by sonication and were designed to carry the FP antigen composed of Ag85B-ESAT6-Rv2034 and sized between 80 and 120 nm; results showed that cationic liposomes containing cholesterol increased liposome stability, cellular uptake and dendritic cell activation; cationic lipids improved interactions with dendritic cells and enhanced the Th1-type immune response in a lipid-dependent manner; specifically, 1,2-dioleoyl-sn-glycero-3-ethylphosphocholine (EPC) demonstrated the highest immunogenicity, robust dendritic cell activation and a strong Th1 response measured as the number of CD4+ T-IFNγ^+^, especially when combined with cholesterol, while maintaining moderate cytotoxicity; in contrast, DODAB exhibited higher cytotoxicity but was effective in dendritic cell activation, whereas DOTAP showed moderate uptake and activation, which were enhanced by cholesterol with minimal increase in cytotoxicity [135].

**Figure 6:**
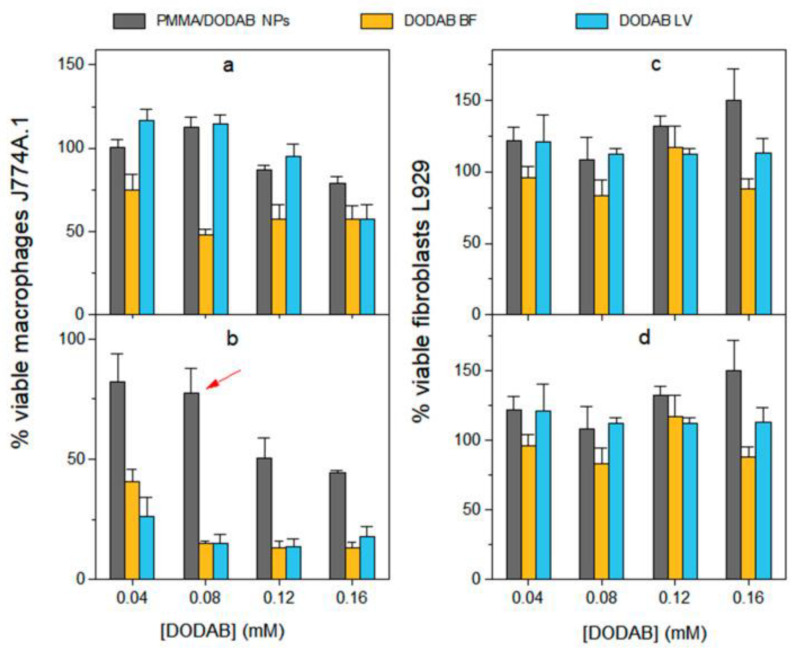
Viability of mammalian cell lines in the presence of cationic adjuvants based on the DODAB cationic lipid as reprinted from reference [16]. J774A.1 macrophages and L929 fibroblasts were exposed to adjuvants PMMA/DODAB (gray bars), DODAB BF (orange bars) or DODAB LV (blue bars) for 3 h (**a**,**c**) or 24 h (**b**,**d**). PMMA is the biocompatible polymer poly (methyl methacrylate). DODAB is the synthetic cationic lipid dioctadecyl dimethyl ammonium bromide, BF is bilayer fragment and LV is large vesicle. The highest cell viabilities in the presence of DODAB were obtained for the PMMA/DODAB adjuvant (gray bars) with a threshold concentration of 0.08 mM DODAB (red arrow).

In our lab, we have been developing strategies to modulate DODAB dose-dependent cytotoxicity over the past decades [16,62,113,136,137]; for doing so we obtained combinations of DODAB with biocompatible polymers such as carboxymethylcellulose (CMC) [137] or poly (methyl methacrylate) (PMMA) [16] which indeed substantially diminished DODAB cytotoxicity against cells in culture measured by the MTT method. In addition, in other formulations, just a thin DODAB bilayer shell surrounded polymeric or inorganic NPs thereby reducing total DODAB dose in comparison to doses employed in formulations with DODAB large vesicles (LV) [138] or liposomes [30]. Importantly, in vaccine design with DODAB, its cytotoxicity against macrophages in culture could be reduced substantially by synthesizing hybrid PMMA/DODAB cationic NPs with excellent immunoadjuvant activity, eliciting balanced Th1 and Th2 responses against ovalbumin as the model antigen [16]. Figure 6 illustrates the cytotoxicity of DODAB as large vesicles (LV), bilayer fragments (BF) or PMMA/DODAB NPs against macrophages and fibroblasts in culture [16].

The enhancement of the BCG vaccine using cationic adjuvants presents a promising strategy to overcome its limited efficacy and provide more robust and long-lasting protection against tuberculosis [139,140,141,142]. Cationic liposomes of DODAB/TDB/cholesterol were used to formulate the fusion protein (FP) HspX-PPE44-EsxV to enhance the efficacy of the BCG vaccine; the addition of cholesterol to these structures contributed to reducing the liposomes’ sizes from 960 nm to 205 nm; the adjuvant effectively stimulated a robust Th1 immune response, evidenced by high levels of IFN-γ and IL-12, which are critical for protective immunity against *Mycobacterium tuberculosis*; additionally, the presence of IL-17 indicated a Th17 response, important for granuloma formation and neutrophil migration; mice primed with BCG and boosted with the DODAB/TDB/cholesterol/FP formulation exhibited the strongest immune responses, demonstrating that this strategy significantly enhanced BCG’s efficacy; the high IgG2a/IgG1 ratio further confirmed the induction of a cellular immune response, underscoring the potential of this adjuvant and antigen combination to provide superior protection against tuberculosis compared to BCG alone [139].

In another attempt to improve the efficacy of the BCG vaccine, DODAB liposomes containing imiquimod (IMQ) a TLR7/8 agonist were employed to carry the FP DR2 composed of two antigens, Rv0572c and Rv3621c targeting different stages of TB infection; the DODAB/IMQ/DR2 vaccine induced strong CD4+ Th1 cell responses and robust IFN-γ+ CD4+ effector memory T-cells (TEM) in mice; the vaccine also resulted in high levels of serum antibodies and cytokines, particularly IL-2+, CD4+, and CD8+ central memory T-cells, indicating sustained immunity over 18 weeks; in vitro challenge experiments showed that splenocytes from vaccinated mice exhibited significant protection against *M. tuberculosis*, with lower bacterial loads compared to control groups [141]. Usually, DODAB LV and liposomes are unilamellar and large varying in size from 500 to 1500 nm [30,138,143,144,145]. Therefore, they will not be directly drained to the lymph nodes and rather stimulate macrophages in situ at the injection site; they are micrometric in size.

DNA and mRNA vaccines represent innovative approaches in the fight against tuberculosis, leveraging genetic information to elicit robust immune responses. Cationic nanoparticles are particularly suited for these vaccines as they enhance the delivery and stability of nucleic acids, thereby improving cellular uptake and immune activation.

Cationic liposomes of DODAB (480 nm in mean size) combined with two pattern recognition receptor agonists: monophosphoryl lipid A (MPLA) and trehalose 6,6′-dibehenate (TDB) were used to deliver a recombinant DNA antigen expressing the fusion protein CMFO, a recombinant fusion protein composed of four multistage antigens from *Mycobacterium tuberculosis*: CFP10, MPT64, Ag85B, and HspX; the inclusion of MPLA and TDB in the DODAB liposomes decreased the zeta potential but increased storage stability, allowing a slower and longer-lasting release of DNA compared to DODAB liposomes alone; mice vaccinated with DODAB/MPLA/TBD/CMFO showed significantly higher levels of antigen-specific T-cells and Th1-biased responses, including higher levels of IFN-γ and TNF-α, compared to DODAB/CMFO and CMFO alone; DODAB/MPLA/TBD/CMFO formulation provided enhanced and persistent protection against *Mycobacterium tuberculosis* aerosol infection, comparable to that of the BCG vaccine [145].

The IC31 adjuvant, composed of the cationic peptide KLKL_5_KLK and the immunostimulatory oligodeoxynucleotide ODN1a, were employed to formulate the Ag85B-ESAT-6 fused antigen; the formulation Ag85B-ESAT-6/IC31 showed significant protection in mice and guinea pigs in an model of pulmonary tuberculosis; Ag85B-ESAT-6/IC31 induced strong antibody production in both BALB/C and C57BL/6 mice; it was also confirmed that the formulation elicited high levels of IFN-γ and low amounts of IL-4, IL-5, and IL-10, further supporting a Th1-biased response. Mice vaccinated with Ag85B-ESAT-6/IC31 showed a significant decrease in infection in the lungs compared to control mice and the vaccinated mice with Ag85B-ESAT-6 in Alum [146]. Against tuberculosis, it is essential to elicit a strong Th1 response because the infective agents are located inside the host cells.

The pressing challenge of developing a universal influenza vaccine lies in overcoming the limitations of current vaccines, which often fail to provide broad-spectrum protection due to the high antigenic variability of influenza viruses. To address this issue, an influenza universal vaccine based on the fused antigen NM2e was developed. To this end, the novel cationic adjuvant formulation composed of DODAB and poly (lactic acid) (PLA) was used. The DODAB/PLA NPs were synthesized through a nanoprecipitation method, resulting in uniformly sized particles of 151 nm with a zeta potential of +30 mV. The NM2e@DODAB/PLA cationic nanovaccine exhibited a fourfold increase in antigen uptake by bone marrow-derived DC compared to the antigen alone, accompanied by robust activation and maturation of DC, characterized by elevated expression of markers such as CD40, CD86 and MHC II, as well as increased production of cytokines including IFN-γ, TNF-α and IL-6. In vivo studies demonstrated the prolonged presence of the antigen in lymph nodes for over 14 days post-immunization. The formulation induced high levels of specific IgG antibodies and strong cytotoxic T lymphocyte activity as indicated by increased secretion of granzyme B and IFN-γ. Most notably, the NM2e@DDAB/PLA nanovaccine achieved over 90% cross-protection efficiency in mice challenged with both homosubtypic (H3N2) and heterosubtypic (H1N1) influenza viruses; NM2e@DDAB/PLA nanovaccine showed with no significant toxicity observed in serum biochemical analyses or histopathological examinations [147]. This formulation for the influenza nanovaccine was based on the concept of combining a biocompatible polymer and a cationic compound as developed earlier in our group for PMMA/DODAB hybrid NPs which also yielded similar results presenting in the model antigen OVA [5,62,113,148].

In another attempt to produce more effective vaccine strategies to induce broad and protective immunity against influenza, DOTAP, a cationic lipid NPs known for its ability to generate potent cytotoxic CD8 T cell responses, was used. DOTAP NPs were prepared and mixed with recombinant influenza hemagglutinin (HA) antigen to form a uniform dispersion. DOTAP NPs significantly enhanced the recruitment of CD4 T-cells secreting IFN-γ and IL-2 and confronted the squalene-based adjuvant AddaVax, even when AddaVax was combined with the TLR9 agonist CpG. DOTAP-adjuvanted vaccine elicited proliferation of cytokine-secreting CD4 T cells in lymphatic organs (LN and spleen), with an approximately 9 to 12-fold increase in IL-2-producing cells and a 5.6 to 6.5-fold increase in IFN-γ-producing cells. The response to HA was broadly distributed across three identified CD4 T cell epitopes, with DOTAP NPs inducing a balanced and robust response to each epitope suggesting that DOTAP NPs generated a comprehensive immune response that is less susceptible to viral antigenic variation [149].

Against malaria, a number of heterologous vectors have been tested using DNA priming and boosting with vaccinia Ankara resulting in high levels of T-cell response [150]. As a major global health threat, malaria particularly affects children in tropical and subtropical regions [151]. The Mosquirix (RTS,S/AS01) vaccine, the most advanced vaccine against malaria, has been approved by the World Health Organization for children in areas of moderate to high transmission but shows variable efficacy of 30–50% and requires multiple doses [152,153]. Despite its breakthrough status, ongoing challenges in vaccine distribution and the need for improved efficacy underscore the importance of continued research and development in the fight against malaria. A self-amplifying RNA (samRNA) encoding *Plasmodium falciparum* reticulocyte binding protein homologue (5PfRH5) was used since this protein is crucial for the parasite’s invasion of erythrocytes. The samRNA was stabilized by DODAB and DC-Chol cationic liposomes, which enhanced delivery and improved the immune response; liposomes were prepared by forming a dry lipid film, rehydrating it in HEPES buffer, and applying sonication to achieve sizes under 100 nm. The liposomes demonstrated high transfection efficiency and low cytotoxicity in vitro using Vero and HEK293T cells with GFP and luciferase reporters; in vivo, intradermal tattooing in mice resulted in significant GFP expression, and immunized mice produced antibodies that recognized native PfRH5 and inhibited *P. falciparum* growth. The use of cationic liposome-stabilized samRNA and tattooing for intradermal delivery is a promising strategy in malaria vaccine development [154,155].

To improve malaria vaccine efficacy, a promising study explored the use of the cationic liposomal adjuvant CAF09, combined with full-length recombinant *Plasmodium falciparum* circumsporozoite protein (Pf rCSP). CAF09 consists of DODAB, monomycolyl glycerol (MMG), and polyinosinic:polycytidylic acid (Poly I:C) and had a diameter of around 400–500 nm [156]. The formulation induced robust antibody and CD8+ T-cell responses, providing comprehensive immunity, and mice immunized with Pf rCSP-CAF09 developed high antibody titers and significant CD8+ T-cell responses, displaying durable sterilizing immunity against transgenic *P. berghei* expressing Pf CSP. The study highlighted the adjuvant’s ability to elicit strong and long-lasting humoral and cellular immunity, addressing limitations of current vaccines primarily inducing antibody and CD4+ T-cell responses [157].

**Figure 7:**
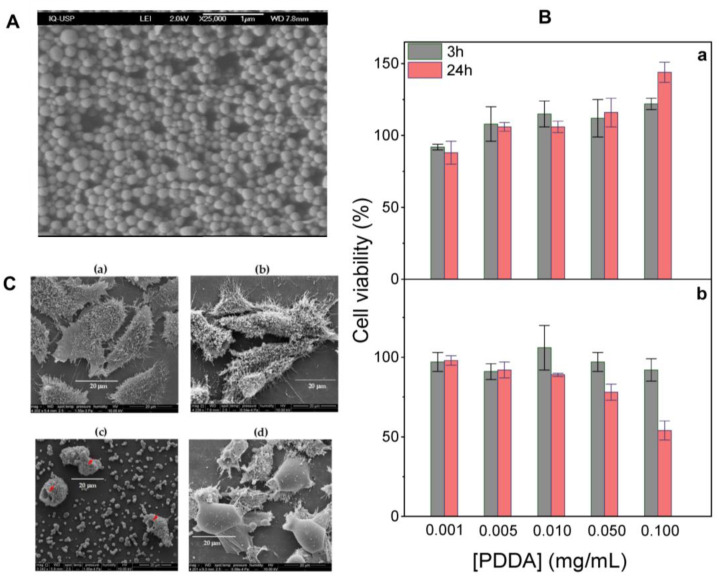
Dose-dependent cytotoxicity of cationic hybrid NPs made of biocompatible polymer and traces of cationic polymer and cationic lipid. (**A**) PMMA/PDDA/DODAB NPs visualized by scanning electron microscopy (SEM) and at 0.56 M methyl methacrylate (MMA) polymerization followed by exhaustive dialysis yielded 5 mg·mL^−1^ PDDA and 2 mM DODAB followed by exhaustive dialysis before use; the bar represents 1000 nm. Reproduced from reference [66]. (**B**) Effect of poly diallyl dimethylammonium chloride (PDDA) concentration in hybrid NPs of biocompatible poly methyl methacrylate (PMMA) on L929 fibroblasts (**a**) or murine macrophages J774A.1 viability (%) after 3 or 24 h interaction (**b**). Reproduced from reference [15]. (**C**) PDDA dose-dependent lysis of fibroblasts in culture; the cells were submitted to increasing PDDA concentrations from (**a**) to (**d**) and are visualized by scanning electron microscopy (SEM) of fibroblasts L929 incubated for 3 h with: (**a**) culture medium; (**b**) 0.01; (**c**) 0.1; (**d**) 1 mg·mL^−1^ PDDA; the red arrows point to holes on the cells at 0.1 mg·mL^−1^ PDDA. Reproduced from [20].

Cationic polymers are another important class of cationic materials used for preparing cationic nanoparticles, though their application is often limited by dose-dependent cytotoxicity, which necessitates careful dose minimization and optimization [20,89,136,158,159]. These polymers readily interact with oppositely charged proteins, facilitating their effective combination and delivery. Figure 7 illustrates the dose-dependent cytotoxicity of the cationic polymer PDDA against fibroblasts in culture and the use of biocompatible PMMA NPs hybridized with PDDA to circumvent its toxicity as reproduced from references [15,20,66]; in this case, the combination of the cationic polymer with the biocompatible polymer produced the desired biocompatibility.

**Figure 8:**
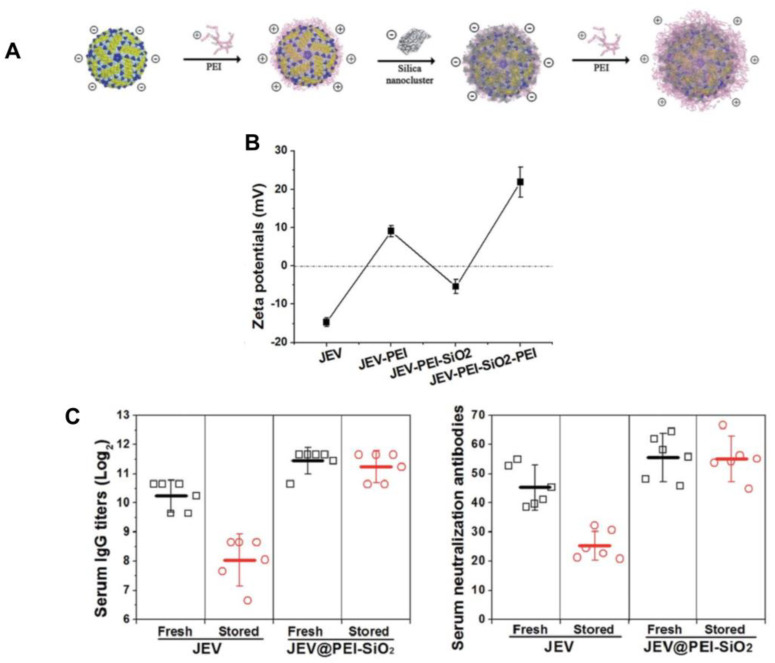
Thermostability of the vaccine conferred by the layer-by-layer cationic assembly of PEI-SiO_2_-PEI on Japanese encephalitis virus (JEV). (**A**) Scheme showing the coating of PEI and silica on the surface of the viral nanoparticles. The first coating with PEI (in magenta) is followed by the second coating with silica (in green) and the third coating with PEI (in magenta). (**B**) Zeta potential measurements of JEV and JEV coated with successive layers of PEI, PEI-SiO_2_ and PEI-SiO_2_-PEI. (**C**) Evaluation of the vaccine’s stability after silicification at pH 7.0, demonstrating enhanced resistant to temperature fluctuations. The modified vaccine maintained its efficacy after prolonged storage at room temperature, enabling more flexible vaccine handling and distribution without the need for cold chain logistics. Adapted from [160].

Aiming to improve the critical issue of vaccine thermal instability, which hinders effective vaccination programs in resource-limited regions, a vaccine formulation used layer-by-layer coating of the Japanese encephalitis virus (JEV); polyethyleneimine (PEI) and silica layers improved the vaccine’s thermostability as illustrated by Figure 8. The vaccine retained its potency at elevated temperatures (up to 42 °C) for extended periods of time; the NPs, averaging 100 nm in diameter, demonstrated a robust thermal protective effect by forming a hydration layer that prevented thermal degradation, and elicited strong immune responses (high serum IgG and neutralization antibody levels) and sustained cellular immunity depicted from IFN-γ secreting splenocytes. This innovative approach not only enhances the stability and efficacy of vaccines but also facilitates their distribution without the need for a cold chain, making it a promising solution for global vaccination efforts [160].

Polymers such as linear PEI and branched PEI were combined with viral glycoprotein gp140 from HIV-1 for immunization; post-immunization, all combinations promoted moderate Th1 responses, intermediate IgG1/IgG2a ratios and significant secretions of Th1 cytokines IL-2, TNF-α, and the pro-inflammatory neutrophiles recruiter GM-CSF, as well as the Th2-associated cytokine IL-5 with all cytokines determined in splenocytes [161].

Approaching the challenge of improving the delivery and immunogenicity of DNA vaccines, specifically against COVID-19, PEI covalently bound to cholesterol (PEI-CHOL), and mannose (PEI-CHOL-MAN) improved cellular uptake, reduced cytotoxicity and targeted mannose receptors on APCs; the synthesis involved conjugating PEI with cholesteryl chloroformate, followed by reacting PEI-CHOL with D-mannose in the presence of sodium cyanoborohydride and acetic acid, resulting in NPs approximately 170 nm and 204 nm in size, with zeta potentials around +15 mV. The PEI-CHOL-MAN NPs demonstrated enhanced cellular uptake and significant stimulation of pro-inflammatory cytokine production, indicating improved dendritic cell maturation and a stronger immune response; the NPs exhibited improved biocompatibility with reduced cytotoxicity and demonstrated stability in various media, maintaining their integrity and protecting the encapsulated DNA from degradation; the incorporation of mannose and cholesterol enhanced targeted delivery, transfection efficiency and safety, making this multifunctional PEI-based NPs formulation a promising approach for enhancing the efficacy of DNA vaccines against COVID-19 and potentially other infectious diseases [162].

PDDA was employed to enhance the delivery and immunogenicity of HIV-1 DNA vaccines using surface-engineered gold nanorods (Au NRs) as adjuvants; in this work cationic molecules such as cetyltrimethylammonium bromide (CTAB), PDDA and PEI were utilized. CTAB, PDDA and PEI were utilized to modify the surface of gold nanorods, creating three distinct formulations: CTAB-Au NRs, PDDA-Au NRs, and PEI-Au NRs. The NPs had similar dimensions, approximately 15 nm × 60 nm, but differed in surface charge and stability, with PDDA- and PEI-modified nanorods showing superior DNA adsorption capabilities and reduced cytotoxicity; PDDA- and PEI-Au NRs significantly improved both cellular and humoral immune responses in vivo compared to naked HIV-1 Env plasmid DNA; these formulations promoted dendritic cell maturation and enhanced T-cell proliferation, indicating a strong immune activation; PDDAC-Au NRs induced a Th2-biased response, characterized by higher IgG1 production, while PEI-Au NRs facilitated efficient DNA release from endosomes due to the proton sponge effect; the strategic modification of gold nanorods not only enhances the transfection efficiency but also provides a stable and biocompatible platform for DNA vaccine delivery; this approach demonstrated that surface-engineered gold nanorods can serve as promising DNA vaccine adjuvants, offering a versatile and effective means to improve the immunogenicity and efficacy of vaccines against HIV-1 [163].

Quaternized polysaccharides have emerged as a promising class of cationic polymers used as adjuvants in vaccine development, enhancing immunogenicity and offering robust protection against various pathogens [164,165]. In an effort to improve the efficacy of the Anthrax Vaccine Adsorbed (AVA), a study explored the use of fucoidan-quaternary chitosan NPs (FUC-HTCC NPs) as adjuvants; NPs, prepared via polyelectrolyte complexation, had sizes ranging from 100 to 300 nm and zeta potentials of ±25 mV; the FUC-HTCC NPs significantly enhanced both humoral and cellular immune responses, leading to higher IgG-anti-protective antigen titers and superior survival rates in mice challenged with the anthrax toxin compared to traditional CpG oligodeoxynucleotides and AVA alone; NPs demonstrated low cytotoxicity, and excellent uptake by dendritic cells, suggesting their safety and efficacy as vaccine adjuvants [166].

To enhance the effectiveness of intranasal influenza vaccines, the use of curdlan-chitosan conjugate NPs (C–O NPs) as mucosal adjuvant was investigated. NPs were synthesized via sonication with an average size of 190 nm with a zeta potential of 16 mV. The C–O NPs demonstrated excellent biocompatibility, while boosting macrophage phagocytosis and the stimulation of DC in vitro. In vivo, mice immunization with the H1N1 subunit antigen and C–O NPs produced significantly higher titers of HA-specific IgG and sIgA, indicating enhanced systemic and mucosal immune responses; this immune activation included the recruitment and activation of key immune cells such as macrophages and T-cells and promoted a balanced Th1/Th2 response, essential for comprehensive protection; the study highlights the potential of C–O NPs to improve vaccine stability and induce robust immunity, addressing the shortcomings of traditional influenza vaccines that often fail to elicit strong mucosal responses [167].

Subunit vaccines are commonly associated with low immunogenicity, necessitating innovative strategies to enhance their effectiveness; addressing this issue, researchers developed a novel carrier system using a complex of 2-hydroxypropyl-trimethylammonium chloride chitosan (HTCC) and amylose to entangle gold NPs (AuNPs) aimed at improving the delivery and immunogenicity of SARS-CoV-2 subunit vaccines; the cationic adjuvants used were HTCC and amylose, which were combined with AuNPs to create positively charged nanocarriers. The NPs were prepared using a modified wet chemical method, resulting in particles with sizes ranging from 33 to 44 nm and a zeta potential of +21.1 mV. These HTCC/amylose/AuNPs could be loaded with high S protein cargo, displayed low cytotoxicity and excellent internalization by RAW 264.7 cells. The immune response was significantly improved, eliciting higher levels of specific IgG antibodies, as well as enhanced T- and B-cell responses in mice compared to the free S protein. This new system offered a stable, efficient delivery method that significantly boosted the immunogenicity of subunit vaccines, making it a promising approach for enhancing vaccine efficacy [168].

Taking advantage of chitosan’s excellent mucoadhesive properties, hybrid DOTAP/chitosan NPs were developed to enhance the effectiveness of intranasal mRNA COVID-19 vaccines; DOTAP formed liposomes via the thin film hydration method, while chitosan was added to improve mucoadhesion by coating the liposomal surface; the optimized nanoparticles, with a size of 360–590 nm and a positive zeta potential of +17 to +24 mV, demonstrated high mRNA encapsulation efficiency (~80.2%) and excellent biocompatibility; the formulation significantly enhanced the immune response by effectively delivering mRNA to the nasal mucosa, leading to high expression levels of the SARS-CoV-2 spike protein in target cells. In mice, this approach elicited strong local immune responses, with high levels of HA-specific IgG and sIgA antibodies in nasal and bronchoalveolar lavage fluids; the chitosan coating ensured prolonged retention and enhanced interaction with the nasal mucosa [169].

In a further effort to enhance the effectiveness of intranasal SARS-CoV-2 vaccines, a study developed mucosal NPs that incorporate mannan, polyarginine and 2′,3′-cGAMP as adjuvants. The NPs’ structure optimized the immune response and delivery efficiency of the domain in SARS-CoV-2 spike protein which binds to the host receptor for infection; the receptor-binding domain (RBD) was chemically conjugated to mannan and then assembled with polyarginine, a cationic polymer, and 2′,3′-cGAMP, a potent stimulator of the STING pathway, through electrostatic interactions. This process resulted in NPs approximately 74 nm in diameter and slightly cationic; RBD-MP-cG NPs demonstrated high mRNA encapsulation efficiency and excellent biocompatibility. Intranasal administration in mice elicited robust local immune responses, with high levels of mucosal IgA and IgG in nasal and bronchoalveolar lavage fluids, and strong systemic immune responses, including elevated levels of RBD-specific neutralizing antibodies. The formulation also promoted a balanced Th1/Th2/Th17 cytokine response, crucial for effective immune protection [170].

Against infectious diseases, comprehensive reviews on nanovaccines have recently appeared [171,172,173,174,175]; however, the importance of cationic NPs is seldom discussed.

## 4. Cationic Nanostructures in Vaccine Design Against Cancer

The rapid growth of tumors necessitates the efficient delivery of nanovaccines to lymphoid organs to promptly stimulate anti-tumor immunity [176]. Optimizing physical properties such as size, charge, colloidal stability and surface ligands can improve the accumulation of nanovaccines in lymphoid organs, such as lymph nodes or the spleen, which is critical for successful cancer immunotherapy. When nanovaccines are administered subcutaneously (s.c.) or intramuscularly (i.m.), they often form localized depots at the injection site, relying on the recruitment and transport of APCs to enrich the LNs. Alternatively, nanoadjuvants delivered via peritumoral, intratumoral (i.t.) or intravenous (i.v.) routes can capture tumor-specific antigens in situ and present them to APCs within the tumor microenvironment or transport them to the tumor-draining lymph nodes (tdLNs) [177]. Additionally, therapies such as chemotherapy, photothermal/photodynamic therapy or radiotherapy can destroy tumor cells, releasing antigens that are subsequently captured by nanoadjuvants and directed to tdLNs, thereby promoting personalized anti-tumor immunity [178].

The spleen, being the largest secondary lymphoid organ, can generate a robust and rapid anti-tumor immune response. Directing nanoparticles to the spleen can enhance the effectiveness of vaccines and cancer immunotherapies, as well as treating intracellular infections such as leishmaniasis, trypanosomiasis, splenic tuberculosis, AIDS, malaria and certain blood-related disorders. However, a macrophage barrier present in the spleen’s red pulp hinders the nanovaccine’s access to T and B lymphocytes. Achieving effective spleen targeting requires precise control over nanoparticle size and the incorporation of surface ligands like albumin to facilitate better accumulation and prolonged circulation [179]. For instance, optimizing the size and coating of nanovaccines post-intravenous (i.v.) administration has proven crucial for improved spleen delivery. Research has demonstrated that clay-based nanovaccines administered intravenously to the mouse spleen triggered potent anti-tumor responses, significantly enhancing both tumor prevention and treatment in lymphoma models compared to conventional subcutaneous (s.c.) vaccination, which typically results in limited anti-tumor efficacy. Nanovaccines formulated using layered double hydroxide (LDH) clay with sizes ranging from 77 to 285 nm and co-loaded with the model antigen ovalbumin (OVA) and the immunostimulatory CpG adjuvant showed that 215 nm-sized LDH promoted the most efficient antigen presentation by dendritic cells. This specific formulation exhibited superior spleen localization and, in vivo, the CO-LDH-215 nanovaccine completely inhibited E.G7-OVA tumor growth in mouse models. When administered via i.v. injection, CO-LDH-215 delayed tumor progression more effectively than s.c. administration, due to the direct and rapid delivery of nanoparticles to the spleen, maximizing the anti-tumor immune response [179].

An obstacle preventing nanovaccines from reaching B or T lymphocytes in the red pulp of the spleen or in the subcapsular sinus of the lymph nodes (LN) is a macrophage barrier [180]. Nanovaccines have to bypass this natural macrophage barrier to interact with the B or T lymphocytes; the maximum achievable delivery efficiency was reported to be only 2% [181]. Improving the delivery efficiency of NPs to solid tumors will possibly be achievable through the appropriate design of multifunctional nanovaccines or through manipulation of biological barriers. In this context, biomimetic NPs disguising their cargo might become very important.

Effective cancer immunotherapy was achieved from nanovaccines based on an injectable hydrogel of graphene oxide and polyethylenimine; this hydrogel protected its loaded cargo of ovalbumin mRNA plus adjuvants from degradation and was directly delivered to lymph nodes. With only one treatment, there was a significant increase in antigen-specific CD8^+^ T cells and the inhibition of tumor growth plus the production of an antigen-specific antibody in the serum preventing the occurrence of metastasis [182].

Cancer immunotherapies seldom produce favorable outcomes in patients since most types of cancer can suppress antigen presentation, dendritic cell maturation and/or activation and infiltration of T lymphocytes in the tumor [183,184].

Nanomaterials have been reported to trigger immunogenic cell death (ICD) in tumors; dying cancer cells then release tumor antigens, pro-inflammatory cytokines (e.g., IL-1β and IL-18) and damage-associated biomarkers (e.g., calreticulin, heat-shock proteins, adenosine triphosphate) leading to APCs’ maturation plus activation and infiltration of T-cells in tumors. Several nanomedicines with ICD-induced anticancer immunity have also major disadvantages associated with toxicity in vivo; the nanomaterial has to be biodegradable and toxicity in vivo has to be reduced [185].

Nanomaterials damaging lysosomes not only hamper lysosomal function of intracellular digestion but also release hydrolytic enzymes such as cathepsins, thereby triggering ICD. The disruption of intracellular clearance and the leakage of lysosomal contents can trigger cell death by a variety of routes such as apoptosis, necroptosis and others [186].

Polycations such as high-molecular-weight PEI (hwPEI) are well-known for cell cytotoxicity due to lysosome rupture often explained by the proton sponge effect since protonation of PEI amine moieties supposedly increases lysosomal pH [187]. Using a nanoparticle pH sensor for pH measurements inside endosomes or lysosomes, determinations of lysosomal pH as a function of PEI content did not detect lysosomal pH changes induced by PEI as previously suggested and shed some doubt on the mechanism behind PEI-induced endosomal escape [188].

In our experience with PDDA, during the dialysis of PDDA samples, we observed an influx of water to the dialysis bag revealing that the polymer induced an increase in osmotic pressure inside the bag. (Indeed, PDDA is a very hydrophilic polymer.) In Figure 7C, we showed the devastating concentration dependent effect that PDDA could cause by disrupting basically all compartments inside cells. Would the effect of PEI polymer be similar to the one observed for PDDA? The increased influx of water in accordance with increases in the osmotic pressure due to the polymer enclosed in the lysosome would be a simple mechanism to explain lysosomal swelling and rupture culminating in cross-presentation of antigen carried by the polymer due to its presence in the cytosol. Furthermore, PEI-mediated cytosolic delivery of oppositely charged antigens occurs by endosomal bursting. For HeLa cells incubated with fluorescent polyplexes, after internalization of the poly (ethylene imine) (PEI) polyplexes in the endosomes, endosomal bursting took place releasing their fluorescent cargo into the cytosol; thereafter, the fluorescent cargo rapidly accumulated in the nucleus [70]. In order to activate potent cellular immune responses, cationic cancer nanovaccines should not only promote improved cellular uptake by DC but also lysosome escape, triggering cross-presentation to activate potent cellular immune response Th 1. The self-assembly of low molecular weight PEI into nanoparticles remarkably enhanced lysosome rupture, cell cytotoxicity and immunogenic cancer cell death [187].

**Figure 9:**
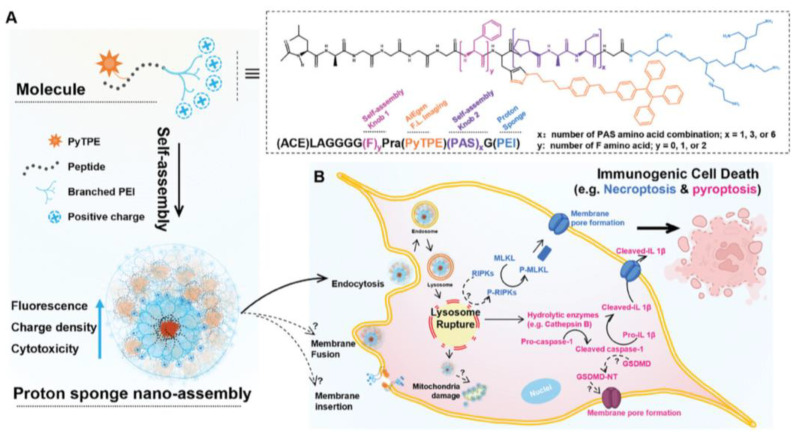
Proton sponge cationic nano-assembly (PSNA) as inducer of cancer cell death. (**A**) Chemical structure of PSNA monomer and its self-assembly into a fluorescent nanostructure. (**B**) Endocytosis of PSNAs by cancer cells triggering cell death and releasing pro-inflammatory cytokines (e.g., IL-1β) known to induce anticancer immunity. The dashed arrows represent suggestions of other possible mechanisms for the internalization of the nano-assembly and the cell. Reproduced from [189] with permission from John Wiley and Sons under a Creative Commons License.

High molecular weight cationic polymers taken up by lysosomes, such as branched poly (ethylenimine) (PEI) or PDDA, are excellent at rupturing lysosomes leading to cell death due to the overt cytotoxicity of the cationic polymers. However, in general, the cytotoxicity of cationic lipids or polymers is dose-dependent [16,20,136]. In the case of tumors that do not respond to tumor antigens, an emerging strategy has been turning a nonresponsive tumor to a responsive one by using nano-assemblies exerting a similar effect as high-molecular-weight branched polyethylene imine (PEI) for rupturing lysosomes, but displaying controllable surface charge density and cell cytotoxicity [189]. These nano-assemblies were constructed from low-molecular-weight branched PEI covalently bound to self-assembling peptides labeled with fluorescent tetraphenylethene pyridinium (PyTPE). The self-assembling of the peptide-PyTPE enhanced surface positive charges and cell cytotoxicity of the nano-assemblies thus resulting in high fluorescence, positive surface charge, cell uptake and cancer cell cytotoxicity. Tumor cells undergoing nano-assemblies triggered death-activated immune cells against the many antigens released by the dead cancer cells, eliciting anticancer immunity [189].Figure 9 illustrates an example of self-assembled cationic nanostructures able to trigger immunogenic cell death (ICD) as reproduced from reference [189].

Immunotherapeutic agents commonly employed for cancer treatment are either antibodies, polypeptides or nucleic acids [190]; liposomes can protect both their hydrophobic or their hydrophilic cargos due to their two versatile compartments: the inner aqueous core and the hydrophobic microenvironment in the closed bilayer core. Their multifunctional potential has been reviewed [191]. Cationic nanoliposomes as carriers can incorporate many functionalities: stability enhancers such as polyethylene glycol moieties, Toll like receptors enhancers such as CpG oligonucleotides, fusogenic peptides, a variety of immune stimulators and/or antigens, glycolipids, lipopeptides, etc. [191].

Although excellent reviews on requirements for successful nanovaccines for cancer immunotherapy are available [192,193], just a few highlight emerging cationic nanovaccines [14,79,184,191,194]. During the past decades, cationic lipids or polymers in NPs have been recognized as potent inducers of antigen-specific T-cells; cationic nanoparticles have offered clinically applicable vaccine formulation platforms [79]. For example, nanoparticles of the block copolymer poly (ethylene glycol)-block-poly(lactic-co-glycolic acid) (PEG-b-PLGA) and cationic lipid packaged mRNA molecules, induced efficient internalization and translation in DC, yielded potent CD8+ T-cell activation and induced significant antitumor effect on the aggressive E·G7-OVA lymphoma model [195].

In another instance, fluoroalkane-grafted poly ethylenimine (F-PEI) for intracellular tumor antigen-encoding mRNA delivery activated the Toll-like receptor 4; thereby, association of F-PEI and the tumor antigen-encoding mRNA, without additional adjuvants, could induce DC maturation, efficient antigen presentation and an anti-tumor immune response. As a cancer nanovaccine, OVA-encoding mRNA in this F-PEI-based adjuvant retarded the growth of B16-OVA melanoma; in combination with immune checkpoint blockade therapy, the F-PEI-based MC38 neoantigen mRNA vaccine eliminated MC38 colon cancer and prevented tumor return after a period of remission [196].

**Figure 10:**
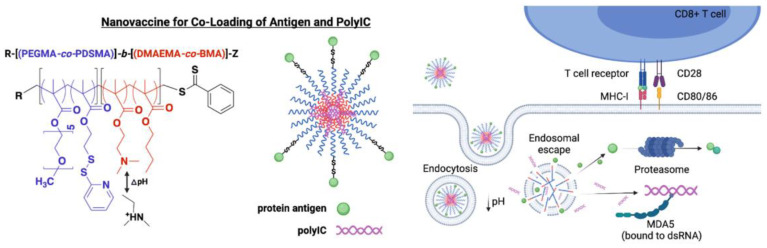
Cationic nanoparticles self-assembled from the sophisticated di-block copolymer construction shown on the left. Combining PEI moiety, antigen and poly-IC, this cancer nanovaccine was able to elicit cross-presentation and efficient CD8+ T cell proliferation. Reprinted from reference [197]. Copyright (2024), with permission from Elsevier.

The electrostatic attraction between PEI cationic moiety in a diblock co-polymer and polyinosinic-polycytidylic acid (poly-IC) facilitated dual endosomal escape of antigen and poly-IC to the cytosol. There was antigen cross-presentation, expression of co-stimulatory molecule and DC cytokine secretion all enhancing the CD8+ T cell responses relative to antigen/poly-IC only; in addition, tumor growth was inhibited in a mouse tumor model [197]. The cationic, co-polymeric NPs could carry and deliver antigens and adjuvants. The co-polymer chemical structure corresponded to a poly (ethylene glycol)-rich first block with reactive moieties for covalent conjugation of antigen using disulfide bonds, and a PEI moiety responsive to pH as a second block for the electrostatic packaging of nucleic acids and facilitated antigen endosomal escape to the cytosol. These NPs promoted endosomal rupture and cytosolic co-delivery of poly-IC and protein antigen. Figure 10 illustrates this approach.

The synthetic poly-IC is similar to the double-stranded RNA that constitutes the genetic material of viruses binding to pattern recognition receptors; it is often used in the clinic to treat cancer [198].

Poly-IC is an agonist of Toll-like receptor 3 located in the endosomal membrane and melanoma differentiation-associated protein 5 (MDA5) in the cytosol; poly IC protection from RNAse degradation could be achieved from the combination driven by opposite charges with cationic polymers such as (PEI) or poly-l-lysine (PLL); poly-IC complexed with PLL and stabilized with carboxymethylcellulose (poly-ICLC; trade mark Hiltonol^®^) has been evaluated in clinical trials and has been reported as a stimulator of IFN-I secretion and/or an enhanced T-cell response. Poly-ICLC activated MDA-5 more effectively than poly-IC since PLL promoted endosomal escape of poly-IC via the “proton sponge effect” [198].

PEI with guanidine moieties transformed PEI’s cytotoxicity into innate immune activation; poly(phenyl biguanidine)-PEI effectively stimulated dendritic cells, promoted their maturation via the TLR4 and NLRP3 routes, and in vivo promoted increased mouse survival due to inhibition of tumor growth [199].

Antigens from cancer cell lysis or membranes are superior to cancer-associated antigens and have been used as the source of cancer vaccines; their combination with cationic nanoparticles made of biosafe polyphenol molecules and metal ions such as Fe^3+^ or Cu^2+^ had to be covalently linked to a mannose moiety, and the nanoparticle had also to incorporate a CpG stimulator for targeting DCs, thereby eliciting a robust antitumor immune response [200].Recently, advances in this type of cancer vaccines were reviewed [201].

This year, a genetically engineered cancer cell membrane nanovaccine simultaneously overexpressed CD40L stimulator and immune checkpoint PD1 for anti-tumor immunotherapy; the CD40L and antigens from cancer cells membranes stimulated dendritic cell-mediated activation of CTL, while PD1 on cancer cell membranes significantly blocked PD1/PD-L1 interaction, inducing a synergistic stimulation of antitumor immune responses; there was satisfactory prevention in a breast tumor mouse model [202].

A lipid nanoparticle (LNP) imitating high-density lipoprotein was loaded with cancer cell membranes with tumor antigens (LNP-M). This was optimized for lymphatic targeting and DC uptake; there was suppression of tumorigenesis and growth and an augmented therapeutic effect of checkpoint inhibitors, notably on the high-stemness melanoma in mice; three LNP-M with comparable particle size (40 nm) but different surface charges (−30, −5, and +20 mV) were obtained by incorporating cetyltrimethylammonium bromide at different amounts; negatively charged LNP-M exhibited superior LN-targeted capacity than its positively charged counterpart, but positively charged LNP-M promoted the DC uptake of LNP-M, due to increased adsorption of LNP-M on the negatively charged cell membrane of the DCs by electrostatic interaction [203].

Using a promising approach, photothermal therapy (PTT) and immunotherapy were combined for the development of an in situ nanovaccine. Nanoparticles (NPs) from Fe^2+^, CpG (Toll-like receptor 9 agonist), DOTAP (cationic lipid) and polydopamine (photothermal agent) were injected into the tumor and exposed to laser irradiation capturing tumor-associated antigens (TAAs) generated upon post-PTT to form the nanovaccines (NPs@TAAs). These vaccines promoted cross-presentation of TAAs, stimulated adaptive immune responses and developed tumors responsive to immunotherapy. As a result, in situ nanovaccines highly improved survival rates and elicited a durable immune memory that prevented metastasis, illustrating the synergism for combined PTT and immunotherapy [204].

Interestingly, the self-assembly of the cationic antimicrobial peptide α-melittin yielding nanoparticles evolved as a simple and effective approach against tumor cells; melittin kill tumor cells and promote the release of whole tumor-cell antigens in situ. Furthermore, α-melittin-NPs drained into LNs and activated macrophages and DC after s.c. injection. After the priming and activation of the effector T-cell response against whole-tumor antigens in LN, the activated effector T-cells reached the tumor, killing the target tumor cells; α-melittin-NPs also induced the infiltration of innate immune cells, especially NK cells and monocytes [205]. Melittin, a main component of bee venom, is a cationic amphiphilic peptide with a linear α-helix structure that can exert pharmacological effects, such as antitumor, antiviral and anti-inflammatory effects in vitro and in vivo as comprehensively reviewed recently [206]. In fact, the potential of cationic antimicrobial peptides and their assemblies requires further investigations for the benefit of cancer immunotherapy [207,208].

Probiotics such as *Lactobacillus rhamnosus* and *Bifidobacterium longum* coated with lipid membrane could be protected against loss of activity and could thereby achieve better colonization in the colon. The subcutaneous transplant of colon cancer in mice was studied against this probiotic formulation, which showed potent preventive and therapeutic efficacy that could be combined with cancer nanovaccines; probiotics formulation alone efficiently reduced the tumor number in the colon besides improving the performance of combinations with cancer nanovaccines [209].

Cancer nanovaccines have been reviewed from several points of view recently, emphasizing types of nanovaccines and carriers, ideal immune responses, factors determining their efficiency, appropriate antigens and many other important characteristics [201,210,211].

In this review, we presented and discussed the main trends regarding emerging cationic nanovaccines and their potential against infectious diseases and cancer. Table 1 summarizes current approaches involving cationic nanovaccines presenting antigens from infective pathogens or cancer cells. Curiously, in Table 1, one can find cationic adjuvants of very different sizes and with the same composition as the DODAB bilayer fragments of about a 70 nm mean diameter and the DODAB large liposomes of about 400 nm mean diameter that yielded the same Th1 biased immune response while carrying the same antigen of *Mycobacterium leprae* [18,26]. In addition, supported cationic DODAB bilayers on polystyrene nanospheres of about 300 nm mean diameter, representing a system of very low polydispersity, also yielded Th1 immune responses while carrying antigens from *Taenia crassiceps,* but in this case, the parasite antigen determined also a Th2 humoral response [19]. Therefore, neither size nor polydispersity appear to matter over this range of particle sizes.

## 5. Conclusions

Cationic compounds such as lipids, surfactants, polymers, peptides, metals and metal oxides all have been tried as adjuvants for vaccines either alone or in interesting hybrid assemblies with other materials, some of them biocompatible. Among all possibilities, the simple ones avoiding covalent binding and depending only on interaction forces between antigen, immune response stimulator and adjuvant seem to be more feasible and straightforward for scaling up in the biotechnology industry. One of the requirements for applications of cationic nanoadjuvants in vaccinology is the systematic study of cytotoxicity. However, in certain instances, precisely this cytotoxicity can be used to disrupt cancer cells in situ. This rupture of cancer cells releases multiple antigens prone to local and systemic presentation to APCs by the novel cationic nanoadjuvants. As a consequence, a strong antigen-specific cellular immune response fights cancer.

## Figures and Tables

**Table 1:** Some examples of emerging cationic nanovaccines for prevention and/or treatment of infectious diseases and/or cancer.

Year	Carrier/Adjuvant	Antigen(s)	Mean Diameter/nm	Immune Response	Ref.
**1997**	DODAB ^1^ liposomes	18 KDa protein *M. leprae*	400	Th1	[26]
**2005**	DODAB/TDB ^2^	Ag85B–ESAT-6 fusion protein from Mycobacterium tuberculosis	485	Th1/Th2	[30]
**2007**	PSS ^3^/DODAB NPs	Taenia crassiceps antigens	309	Th1/Th2	[19]
**2009**	DODAB BF ^4^	BSA 18 kDa protein from *M. leprae*	82 106	Th1	[18]
**2012**	DODAB BF	OVA	274	Th1	[21]
**2012**	PEI ^5^	Plasmidial HIV DNA	250	Th1/Th2	[84]
**2012**	Gold nanorod/PEI	Plasmidial HIV DNA	800	Th1/Th 2	[163]
**2018**	DODAB BF	Outer membrane vesicles Neisseria meningitidis B	160	Th1/Th2	[28]
**2020**	PDDA ^6^	OVA	165	Th2	[20]
**2020**	DOTAP ^7^, DOPE ^8^ and DSPE-mPEG2000 ^9^ Cationic lipid NPs	mRNA of hemagglutinin from influenza H1N1	200	Th1/Th2	[105]
**2021**	PMMA ^10^/DODAB/PDDA NPs	OVA	225	Th1/Th2	[15]
**2021**	PMMA/DODAB NPs	OVA	81	Th1/Th 2	[16]
**2023**	PLGA ^11^/polyamidoamine dendrimers	AD5-TRP2 antigen from melanoma	150	Th1	[104]
**2023**	Gold/amylose NPs	S protein from coronavirus	35	Th1/Th2	[168]
**2023**	Poly arginine/mannan/STING ^13^ NPs	Receptor binding domain of the S protein	100	Th1/Th2/mucosal immunity	[170]
**2024**	Curdlan sulfate/O-(2-hydroxyl) propyl-3-trimethyl ammonium chitosan chloride conjugate NPs	Hemagglutinin from H1N1 influenza virus	191	Th1/Th2/mucosal immunity	[167]
**2024**	Cationic nanoliposomes	Ag85B-ESAT6-Rv2034 (AER) fusion protein from Mycobacterium tuberculosis	150	Th1	[135]
**2024**	PLA ^12^/DODAB NPs	NM2e antigen (neuraminidase from influenza virus)	151	Th1/Th2	[147]
**2024**	Fluorescent label/peptide/branched PEI self-assembled NPs	Tumor cells antigens	25	Immunogenic tumor cell death	[189]
**2024**	Poly guanidine-PEI NPs	OVA	200	Immunogenic tumor cell death	[199]

^1^ Dioctadecyldimethylammonium bromide; ^2^ trehalose 6,6′-dibehenate; ^3^ Polystyrene sulfate; ^4^ Bilayer fragment; ^5^ Polyethyleneimine; ^6^ Poly diallyldimethylammonium chloride; ^7^ 1, 2-dioleoyl-3-trimethylammonium-propane; ^8^ 1, 2-dioleoyl-sn-glycero-3-phosphoethanolamine; ^9^ 1, 2-distearoyl-sn-glycero-3-phosphoethanolamine-N-(methoxy (polyethylene glycol)-2000); ^10^ Poly methyl methacrylate; ^11^ Polylactic-co-glycolic acid; ^12^ Poly lactic acid; ^13^ Stimulator of Interferon Genes.

## Data Availability

Not applicable.
